# Surgical Intervention for Pectoralis Major Muscle Rupture: Report of Acute and Chronic Cases

**DOI:** 10.1155/2018/2380241

**Published:** 2018-11-04

**Authors:** Erica Kholinne, Cheong-su Lim, Julius I. Kho, In-Ho Jeon

**Affiliations:** ^1^Department of Orthopedic Surgery, Asan Medical Center, University of Ulsan College of Medicine, Seoul, Republic of Korea; ^2^Department of Orthopedic Surgery, St. Carolus Hospital, Jakarta, Indonesia

## Abstract

Pectoralis major muscle rupture is becoming more frequent due to the current trends toward high-contact sports. We reported 2 cases with acute and chronic injury settings along with the strategy to treat each of it.

## 1. Introduction

Pectoralis major muscle rupture is increasing in incidence due to higher interest in high-contact sports in the last decade [1]. Delayed diagnosis or misdiagnosis can predispose to its management. Acute cases mostly will require only direct repair. Delayed diagnosis which leads to a chronic setting in some cases can be repaired without the use of graft, but some will require reconstruction by using graft [2]. We present 2 cases of pectoralis major rupture (acute and chronic injury) treated surgically in our center.

## 2. Material and Methods

### 2.1. Case 1

**Figure 1:**
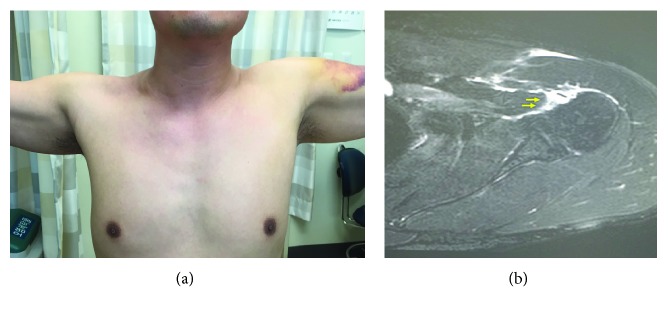
(a) The left shoulder presented with loss of axilla fold contour and bruises over the upper arm. (b) Total rupture of the pectoralis major at humeral insertion showed at T2-weighted axial MRI of the left shoulder.

A 44-year-old male fell backward with his left arm supporting his body weight. His left shoulder was forced to rotate externally and hyperextended. A sudden axilla pain was felt. At physical examination, bruises were noted along with loss of pectoralis major contour (Figure 1(a)). Active shoulder forward flexion was 160° and external rotation was 70°. The internal rotation was measured to be at the Th 12 level, compared to the Th 7 level of the opposite side. There was also a decrease of internal rotation power. Sensory distribution was unaffected. There were no significant findings on plain radiograph. Magnetic resonance imaging (MRI) confirmed a complete rupture of the clavicular head, pectoralis major insertion with mild retraction (Figure 1(b)).

#### 2.1.1. Surgical Technique

**Figure 2:**
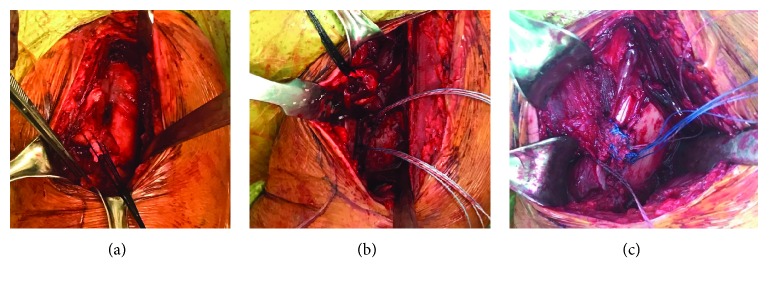
(a) Mobilization allowed direct repair fashion; (b) two bone anchors fixed at footprints; (c) double Krackow stitches were applied to the musculotendinous junction and limbs were parachuted down and tied.

Surgery repair was performed 1 week after the injury. A routine deltopectoral approach was used. Blunt dissection revealed a complete rupture of the clavicular head. Tendon was mobilized over stay sutures with respect to lateral pectoral neurovascular bundles. A trial of reduction was made at the lateral head to the long head of biceps (Figure 2(a)). Two double-loaded 4.5 mm bone anchors (HEALICOIL PK suture anchor, Smith and Nephew, US) are placed in the footprint 1 cm away towards another in a divergent trajectory following decortication (Figure 2(b)). A double Krackow grasping suture was done with one limb of the pair suture. The contralateral limb was pulled to push the tendon down to the footprint. Standard surgical knots were tied in 45° arm abduction (Figure 2(c)). Postoperatively, a sling was used for 1 week. Shoulder exercise was restricted to passive assisted motion only. Assisted motion was started at 3 weeks and progressed to active motion at 6 weeks postoperatively. At 1-year follow-up, the patient returned to preinjury level function as a recreational tennis player with no complaint on the affected extremity during games.

### 2.2. Case 2

**Figure 3:**
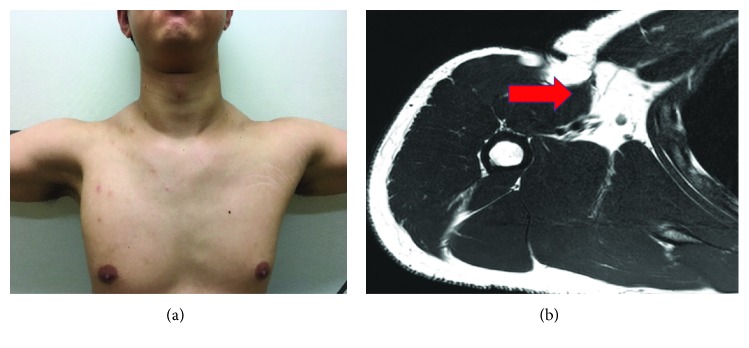
(a) The right shoulder presented with loss of axilla fold with no bruises due to chronic presentation. (b) T2-weighted images of MRI showed complete torn of pectoralis major muscle in the osteotendinous insertion with retraction up to the medial border of the anterior margin of the deltoid muscle.

A 26-year-old male presented with persistent right shoulder pain and weakness after falling down during a jujitsu sparring 7 months ago. The patient declined MRI due to the normal radiograph of the shoulder. On serial examinations, the anterior axillary fold was obliterated (Figure 3(a)). The range of motion (ROM) of the shoulder was full with 4/5 weakness on adduction and internal rotation. Weakness persisted for another 3 months which necessitate MRI concluding pectoralis major rupture with retraction to the medial border of the deltoid muscle (Figure 3(b)).

#### 2.2.1. Surgical Technique

**Figure 4:**
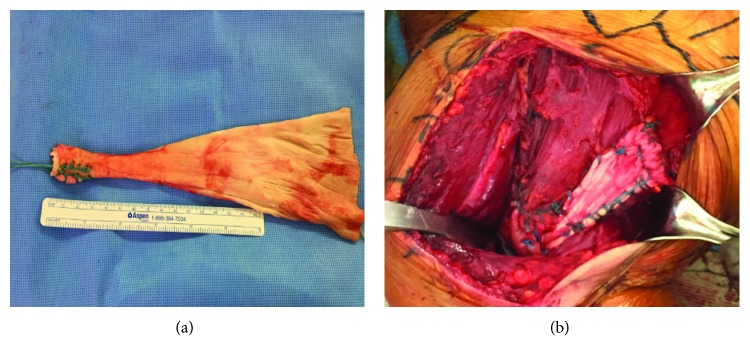
(a) A 20 cm Achilles tendon allograft was prepared according to defect size. (b) Sutures on the anchors are then attached to the allograft-folded end in a modified Mason-Allen suturing technique.

Surgical dissection revealed that the sternal head and clavicular head were retracted medially. It was noted that tendon could not be pulled adequately to the insertion site; therefore, reconstruction was preferred over a repair. Two double-loaded 4.5 mm suture anchors (HEALICOIL PK suture anchor, Smith and Nephew, US) were placed 15 mm apart on footprint. A 20 cm Achilles tendon allograft was prepared and folded once at approximately 7 cm from its distal tapered end (Figure 4(a)). The distal free end was attached with ETHIBOND 2 to the clavicular head while the proximal free end was attached to the sternal end in Krakow suturing technique, approximating with tensionless construct upon attachment to the insertion site. Sutures on the anchors are then attached to the allograft-folded end in modified Mason-Allen technique (Figure 4(b)). Postoperative protocol was similar to the first case. At 1-year of final follow-up, the patient returns to preinjury level function with no complaint on the affected extremity during sports activity.

## 3. Discussions

Pectoralis major injury is usually resulted from indirect injuries that are associated with weight training activities such as weight lifting and bench press. The use of anabolic steroid for individuals with vigorous strength training may increase the disproportionate strength of muscle to tendon at the musculotendinous junction and of the insertional site, making these tendons more susceptible to injury [2]. Diagnosis of complete or incomplete pectoralis major injury may present a clinical dilemma in the acute phase of the injury [3]. This is due to the presence of swelling together with the pain of the shoulder. Total rupture is infrequent because of the complex anatomy of the tendon. The sternal head lies deep and proximal to the clavicular head. This is the reason that clinical diagnosis in the acute phase is quandary because of the possibility of the intact clavicular head [4]. Isolated rupture of the clavicular head is underreported. We reported an isolated clavicular head rupture in an acute setting which is uncommon.

In case of total rupture, surgical treatment is advisable. The results of the surgical procedure are relatively good despite variation in technique and rehabilitation protocol [5]. Delay in either diagnosis or treatment may result in chronic case which may challenge the surgeon's skill to have an optimum bone to tendon contact resulting on the need for graft reconstruction [6]. We reported 2 cases, acute injury treated with early surgical repair and chronic injury treated with allograft reconstruction. Any complete rupture of any heads involving either the myotendinous junction or enthesis site should be treated surgically. In a meta-analysis by Bak et al., 88% of the patients treated surgically had excellent/good results while only 27% of those treated conservatively achieved excellent/good results [7]. That study also showed that 57% of those surgically treated within eight weeks of injury obtained excellent results compared to 16% who had delayed surgical repair. Surgically treated patients also returned to 99% peak torque of the uninjured upper limb while those treated conservatively only achieved 56% of the uninjured side. We chose surgical repair for an acute young patient to restore his strength and achieve maximum functional outcome. Studies had shown that early repair within 8 weeks of injury will provide optimal result. Delayed surgery is more technically demanding and result is less predictable [7–9]. Delayed surgical repair will only reveal favourable results if retracted torn muscle was limited by the adhesion of the injury zone or the existence of the intact head.

There are many surgical repair techniques that had been reported. Most surgical repairs are similar in a form of trough-drill-and suture and differ only in fixation manner. Options of fixation can vary from transosseous heavy sutures, periosteal sutures, cancellous screws and washers, staples, and anchors [8, 10–13]. The attempt of reinforcing or augmenting may follow the repair procedure [11]. We used the anchors for fixation considering that our case is acute; stump was able to mobilized without any tension and good bone quality of the patient. Advantages for using anchor construct include shorter surgical duration and less soft tissue stripping. Nonetheless, the issue over local host reaction to both metal or biodegradable anchors and smaller bone to tendon contact area is inevitable [14]. Study has shown no difference between transosseous repair and anchor repair in regard to the ultimate failure and stiffness [14, 15]. There are benefits and disadvantages to both repair constructs. Transosseous repair as a gold standard has been shown to have good long-term outcomes. It allows larger bone to tendon contact area. Nevertheless, tendon shortening is unavoidable in this construct for the need to be pulled into the trough. Another downside of this construct is that the sutures tied over the bony bridge could fail [14]. We did not augment our surgical repair due to the inadequate length of the remaining tendon to be oversewed on it. In our first case, the sternal head remained intact; hence, footprint identification is not difficult since the existence of sternal head lamina. We used Krackow suture technique for grasping of the stump because the torn stump was found to be of a single layer [16].

Misdiagnosis and delay in presentation are often found. Study has reported that patients may decline initial surgical attempt despite affirmative diagnosis [6]. In chronic complete tears, the presence of muscle retraction and reduction of remaining tendon often hinder direct surgical repair. As our case, the patient attempted to be in the preinjury state and is limited by functional inability. Thus, there is prompt request for surgical treatment. To our knowledge, only a small number of delayed surgical cases have been reported previously [1, 2, 17–19]. Surgical reconstruction usually amends for larger incision to facilitate better dissection and tendon mobilization [20]. Large amounts of adhesed scar tissue from the injury zone might mask the torn tendon as an intact unit. Careful dissection should be taken to delineate the injury pattern. In our case, we performed a combination of digital blunt and sharp dissection for adhesiolysis and stump mobilization. Unfortunately, the mobilized stump did not meet the tensionless criteria to be repaired primarily. We strongly recommend that tensionless surgical repair is important for better outcome; hence, reconstruction took place for the second case. We also would like to emphasize that reconstruction is preferred when primary repair was not possible due to chronicity of the tear. Pectoralis major reconstruction with graft was chosen in order to obtain a stable repair allowing early functional rehabilitation but without excessive tension. The background of having an allograft rather than autograft reconstruction is to avoid the morbidity of the donor site, to reduce intraoperative time of graft harvesting, and to avoid the shape incompatibility between the graft and recipient site. Because of the absence of tendon remnant, the anatomic footprint was determined according to a previous cadaveric study, approximately 5 cm away from greater tuberosity at the bicipital groove [21]. Graft options may include allograft (Achilles) or autograft (bone-patellar tendon, fascia lata, and hamstring) [1, 2, 17–19]. We have chosen the Achilles allograft which offers several benefits over others. This graft is readily available in our institution. Achilles fan-shaped contour is similar to the pectoralis major. This will facilitate the technical aspect to allow it to directly overlay on to the large pectoralis major. The need for time-consuming and complicated technique for graft preparation (e.g., tubularization, weaving, and cross stitching) is surely inevitable. Another structural advantage is that they both have 2 heads that coalesces in the inferior part in a rotating manner before they insert into the bone with a comparable footprint area [4, 16, 22]. Therefore, this structural similarity will be advantageous for surgeons biomechanically. Last but not least, the longstanding inactive muscle from a delayed repair may influence the outcome of graft reconstruction. Nevertheless, we believe that graft reconstruction may be the only way to bridge the defect between a chronically torn and retracted muscle and its normal humeral insertion. The aim of this reconstruction is to have a stable repair allowing for early functional rehabilitation but without excessive tension.

Our reports have only 2 cases as a limitation. Although our results were favourable, still large, randomized, controlled trial of specific methods is necessary. More importantly, we emphasize the need of serial examination and patient education for overcoming inferior results towards surgical treatment. Ideally, these injuries should encourage acute intervention to decrease the demanding graft reconstruction.

## 4. Conclusions

Rupture of the pectoralis major is rare and mainly occurs in male population. Eccentric muscle loading remains a hallmark of the injury. Both acute and chronic rupture cases share the characteristic feature of losing anterior axillary fold. Early surgery treatment is preferable; nevertheless, good outcomes are achievable in a delayed setting.
